# Perioperative Outcomes and Learning Curve for Robotic Liver Resection: An Exploratory Single-Surgeon CUSUM Analysis Stratified by IWATE Difficulty Score

**DOI:** 10.3390/cancers18172739

**Published:** 2026-08-24

**Authors:** Roberta Vella, Kejd Bici, Sergio Li Petri, Duilio Pagano, Pasquale Bonsignore, Alessandro Tropea, Sergio Calamia, Caterina Accardo, Ivan Vella, Irene Vitale, Federica Chimenti, Marco Barbara, Fabrizio di Francesco, Salvatore Gruttadauria

**Affiliations:** 1 IRCCS ISMETT—UPMC Italy, Via E. Tricomi 5, 90127 Palermo, Italy; rvella@ismett.edu (R.V.); kbici@ismett.edu (K.B.); slipetri@ismett.edu (S.L.P.); dpagano@ismett.edu (D.P.); pbonsignore@ismett.edu (P.B.); atropea@ismett.edu (A.T.); scalamia@ismett.edu (S.C.); caccardo@ismett.edu (C.A.); ivan.vella91@hotmail.it (I.V.); ivitale@ismett.edu (I.V.); fchimenti@ismett.edu (F.C.); mbarbara@ismett.edu (M.B.); fdifrancesco@ismett.edu (F.d.F.); 2 UPMC Italy, 90127 Palermo, Italy; 3 Department of Precision Medicine in Medical, Surgical and Critical Area (MePreCC), University of Palermo, 90127 Palermo, Italy; 4 Department of General Surgery, Milano Bicocca University Hospital, 20126 Monza, Italy; 5 Department of General Surgery and Medical-Surgical Specialties, University of Catania, 95123 Catania, Italy

**Keywords:** robotic liver resection, hepatobiliary surgery, learning curve, CUSUM, IWATE difficulty score, textbook outcome, minimally invasive surgery, hepatectomy

## Abstract

**Background:** Robotic liver resections (RLRs) are rapidly expanding, yet the association between the learning curve, procedural complexity, and outcomes at intermediate-volume centers remains poorly defined. We evaluated the learning-curve trajectory and perioperative outcomes of a single surgeon’s initial RLR experience according to procedural complexity (IWATE difficulty score). **Methods**: We retrospectively analyzed 58 consecutive RLRs at an intermediate-volume center. We stratified outcomes by IWATE difficulty category and chronological tertile (early/middle/late). Textbook outcomes (TOs) and a composite failure endpoint (conversion, major complications [Clavien–Dindo ≥ IIIa] and 90-day mortality) were also assessed. Learning-curve behavior was examined with CUSUM and risk-adjusted CUSUM (RA-CUSUM) analyses. **Results:** Fifty-four procedures (93.1%) were minor resections and four were major hepatectomies; two were classified as IWATE Expert difficulty. Median estimated blood loss was 100 mL; conversion occurred in 10.3%, overall morbidity in 10.3% and severe complications (Clavien–Dindo ≥ IIIa) in 3.4%, with no mortalities within 90 days. TOs were achieved in 69.0% using the Delphi (TOLS) definition and in 46.6% using a length-of-stay-extended definition. Operative time and length of stay increased significantly with IWATE difficulty (*p* < 0.001 and *p* = 0.030), as did the composite failure endpoint (*p* = 0.039), whereas blood loss and complications did not. TOs decreased with difficulty under the extended definition (*p* = 0.016). No outcome except estimated blood loss differed across chronological tertiles (*p* = 0.032). Operative time was associated with the IWATE score (26.3 min per point; R^2^ = 0.32) but not with case order (*p* = 0.93). CUSUM and RA-CUSUM curves showed a non-linear, multiphase pattern without an identifiable inflection point, with extremes attributable to individual high-complexity procedures. **Conclusions:** In this exploratory single-surgeon series, consisting predominantly of minor resections, no case-number threshold could be identified, and perioperative outcomes were more closely associated with procedural complexity than with chronological experience, supporting a complexity-adjusted interpretation of RLR outcomes rather than a fixed case-number learning threshold.

## Simple Summary

Robotic liver resections (RLRs) are rapidly expanding the field of minimally invasive hepatic surgery for both benign and malignant diseases. We conducted a retrospective analysis of consecutive RLRs performed by a single surgeon without prior experience in robotic general surgery, focusing on technical proficiency and perioperative outcomes. Procedures were stratified according to the IWATE difficulty score. No mortality and no post-hepatectomy liver failure occurred, and severe complications were infrequent, without clustering of adverse events in the earliest cases. Operating time, hospital stay and the likelihood of conversion were more closely associated with procedural complexity than with case order, and no case-number threshold could be identified within the 58 procedures analyzed. In a series composed predominantly of minor resections, these exploratory findings suggest that expectations and case selection during the early adoption of robotic liver surgery may be better guided by procedural complexity than by case sequence alone.

## 1. Introduction

Robotic liver resection (RLR) has been reported since the early 2000s and has progressively expanded from minor resections to major hepatectomies and other complex hepatobiliary procedures [1,2,3]. Its main technical advantages over conventional laparoscopy include articulated instruments, enhanced dexterity, tremor reduction, and high-definition three-dimensional visualization, which facilitate fine dissection in confined and complex anatomical spaces [4]. The 2018 expert consensus endorsed the use of robotic liver surgery, affirming its safety and feasibility [5]. However, the pace at which proficiency is achieved during the transition to robotic hepatobiliary surgery remains debated [6]. Reported analyses of operative time do not account for the wide variability in procedural complexity inherent to RLRs [7], with only few papers specifically reporting the effect of robotic logistics and perioperative preparation [8,9]. Additionally, most published series describe surgeons who had already achieved general robotic proficiency in another subspecialty, typically colorectal surgery, before transitioning to hepatobiliary procedures [10]; the learning trajectory of surgeons trained directly in robotic hepatobiliary surgery, without this preceding generalist caseload, remains unexplored.

Most of the available evidence on robotic hepatobiliary learning curves and outcomes comes from a small number of high-volume robotic referral centers, leaving the feasibility and reproducibility of this approach at intermediate-volume centers comparatively underexplored [11]—a question of direct relevance to the broader diffusion of minimally invasive hepatobiliary surgery.

The aim of the present study was to characterize the perioperative outcomes and learning trajectory of a single-surgeon robotic liver resection program in a robotic HPB intermediate-volume center.

## 2. Materials and Methods

### 2.1. Study Design and Setting

This was a retrospective, single-center cohort study of all consecutive patients undergoing robotic liver resection at IRCCS ISMETT—UPMC Italy, between November 2024 and April 2026. All procedures were performed by a single experienced transplant and hepatobiliary surgeon, with an annual caseload of approximately 150 hepatectomies, including 50 minimally invasive liver resections, within the structured implementation of a robotic hepatobiliary surgery program. The study aimed to characterize the feasibility, safety, and learning trajectory of robotic liver resection during the progressive development of the program within an established liver surgery and transplant center.

Procedural difficulty was scored preoperatively for every case using the IWATE system, and intraoperative and postoperative variables were recorded contemporaneously in the institutional prospective database. Outcomes were subsequently compared along two independent axes: procedural complexity, using the four IWATE difficulty categories, and accumulated experience, using chronological tertiles of the case sequence.

### 2.2. Data Collection

Data were extracted from a prospectively maintained database and included patient demographics, comorbidities, tumor characteristics, IWATE difficulty scoring [12], operative details, and postoperative outcomes. Procedural difficulty was scored for each resection using the IWATE difficulty scoring system and categorized into four groups: low (score 1–3), intermediate [4,5,6], advanced [7,8,9], and expert [10,11,12]. Textbook outcome (TO) was assessed using two definitions. The primary definition followed the international Delphi consensus for liver surgery (TOLS) [13] and required all of the following: absence of an intraoperative incident, absence of postoperative bile leak, absence of a severe postoperative complication (Clavien–Dindo grade ≥ IIIa), absence of readmission within 90 days, absence of mortality within 90 days, and an R0 resection margin in oncologic resections. Postoperative complications were graded according to the Clavien–Dindo classification [14]; bile leak and post-hepatectomy liver failure were defined and graded according to the criteria of the International Study Group of Liver Surgery [15,16]. A secondary, extended definition additionally required ≤4 days of length of stay of for minor and ≤7 days for major resections, classified according to the Brisbane 2000 terminology [17]. Length of stay was added because postoperative recovery and discharge efficiency are relevant process measures when assessing the implementation of a new surgical program [18]; because this component is not part of the published consensus, the two definitions are reported separately throughout, and the Delphi definition is used for comparison with published series.

Conversion to open surgery is not part of the consensus TO definition and was not included in either version. However, since conversion may reflect procedural difficulty and surgeon experience [18], an exploratory composite endpoint combining textbook outcome with absence of conversion was defined and analyzed separately under both TO definitions. This composite endpoint is not a variant of the internationally established textbook outcome and is reported as an exploratory measure only.

### 2.3. Statistical Analysis

Continuous variables were assessed for normality using the Shapiro–Wilk test and were summarized as appropriate. Comparisons of continuous variables across groups were performed using the Kruskal–Wallis test, whereas categorical variables were compared using Fisher’s exact test. Trends across chronological tertiles were assessed using the Jonckheere–Terpstra test for continuous variables. All tests were two-sided, and a *p*-value < 0.05 was considered statistically significant. Statistical analyses were performed using Stata, version 17.0 (StataCorp LLC, College Station, TX, USA).

Because the IWATE Expert category comprised only two procedures, a pre-specified sensitivity analysis was performed in which the Advanced and Expert categories were combined.

### 2.4. CUSUM and Risk-Adjusted CUSUM Analyses

Learning-curve behavior was examined with cumulative sum (CUSUM) analysis [19,20] of operative time, estimated blood loss, the composite failure endpoint and textbook outcome, plotted against the chronological case sequence.

For the unadjusted CUSUM, the reference value was the cohort mean of the outcome or the cohort event rate for binary outcomes, and the curve is the running sum of the deviation of each observed value from that single fixed reference.

For the risk-adjusted CUSUM (RA-CUSUM), the fixed reference was replaced by a case-specific expected value predicted from the IWATE difficulty score of that procedure [21]. Both curves start at zero and are plotted against case number. An upward-sloping segment therefore indicates a run of cases performing worse than expected for their difficulty, and a downward-sloping segment better than expected; a change in slope that is sustained identifies a candidate transition point.

The cohort was additionally stratified into chronological tertiles, with trends tested using the Jonckheere–Terpstra test for continuous variables and logistic regression on the ordered period for binary outcomes.

## 3. Results

### 3.1. Patient and Baseline Characteristics

Fifty-eight consecutive RLRs were included. The median age was 65.5 years (IQR: 54–72), and 35 patients (60.3%) were male. The mean BMI was 26.5 ± 5.2 kg/m^2^. Most patients were ASA class III (33, 56.9%), and 42 (72.4%) had at least one comorbidity, with a mean Charlson Comorbidity Index of 4.5 ± 2.8. Liver cirrhosis was present in 17 patients (29.3%); Child–Pugh class was documented in 15 of them and was class A in all. One patient had portal hypertension (1.7%). A prior abdominal operation had been performed in 36.2% (*n* = 21) of patients, and 13.8% (*n* = 8) received neoadjuvant therapy. Full baseline characteristics are reported in Table 1.

### 3.2. Tumor and Operative Characteristics

Hepatocellular carcinoma was the most frequent indication (27, 46.6%), followed by benign/non-oncologic disease (15, 25.9%), colorectal liver metastasis (6, 10.3%), and cholangiocarcinoma (5, 8.6%). Median tumor size was 4.0 cm (IQR: 2.5–7.0), and the vast majority of resections were performed for solitary lesions. The median IWATE score was 5 (IQR: 3–6); 25.9% (*n* = 15) of resections were classified as low difficulty, 55.2% (*n* = 32) as intermediate, 15.5% (*n* = 9) as advanced, and 3.4% (*n* = 2) as expert. Conversion to open surgery occurred in 6 cases (10.3%). None of these were due to intraoperative surgical complications or bleeding in an emergency setting. Full details are reported in Table 2.

The median operative time was 250 min (IQR: 180–360), and median estimated blood loss was 100 mL (IQR: 0–100). No patient required an intraoperative blood transfusion. The Pringle maneuver was used in 48.3% of cases. The median hospital stay was 4.5 days (IQR: 4–6). Three patients were admitted to the intensive care unit. Postoperative complications occurred in six (10.3%) patients, with two Clavien–Dindo grade ≥ IIIa events (one grade IIIa and one grade IIIb; 3.4% overall); bile leak occurred in one patient (1.7%); and no cases of post-hepatectomy liver failure or mortality within 90 days were observed. One patient (1.7%) required reoperation for a postoperative complication. Textbook outcome was achieved in 40 of 58 patients (69.0%) using the Delphi definition and in 27 of 58 (46.6%) using the extended definition including length of stay. The exploratory composite endpoint combining textbook outcome with absence of conversion was achieved in 63.8% and 43.1% of patients under the two definitions, respectively. A composite failure endpoint comprising conversion, severe postoperative complications or mortality within 90 days occurred in 8 patients (13.8%). Detailed perioperative outcomes are presented in Table 3.

### 3.3. Outcomes Stratified by IWATE Difficulty Category

Operative time increased significantly across IWATE categories, from a median of 180 min in the low-difficulty group to 440 min in the expert-difficulty group (Kruskal–Wallis *p* < 0.001). Hospital length of stay also increased with procedural difficulty (*p* = 0.030). Blood loss, postoperative complications and Clavien–Dindo grade did not differ significantly across categories. The composite failure endpoint increased with difficulty, from no event in the low-difficulty group to 12.5%, 33.3% and 50.0% in the intermediate, advanced and expert groups (*p* = 0.039), and conversion showed a similar gradient (0% to 50.0%; *p* = 0.055) and was associated with the IWATE score on logistic regression (odds ratio: 1.72 per point, 95% CI: 1.14–2.59; *p* = 0.010).

Textbook outcome behaved differently under the two definitions. Under the extended definition (including the length of stay), it decreased across categories, from 73.3% (11/15) in the low-difficulty group to 37.5% (12/32) in the intermediate and 22.2% (2/9) in the advanced group (*p* = 0.016). Under the standard Delphi definition, no significant association was present (80.0%, 68.8%, 44.4% and 100%, respectively; *p* = 0.29). Both procedures in the Expert category achieved textbook outcome under both definitions; with only two patients, this figure cannot be interpreted as evidence of better outcomes at the highest difficulty level. In the pre-specified sensitivity analysis combining the Advanced and Expert categories (Low *n* = 15, Intermediate *n* = 32, Advanced/Expert *n* = 11), operative time (*p* < 0.001), length of stay (*p* = 0.001), the composite endpoint including conversion (*p* = 0.012) and the composite failure endpoint (*p* = 0.028) remained significant, whereas the association between the extended textbook outcome and difficulty did not (*p* = 0.064). The results are detailed in Table 4.

### 3.4. Chronological Cohort Comparison

When the cohort was divided into chronological tertiles (early, middle, and late thirds of the case sequence), the IWATE score did not increase significantly over time (Kruskal–Wallis *p* = 0.40; Jonckheere–Terpstra trend *p* = 0.89). Operative time likewise did not show a significant trend (*p* = 0.55; trend *p* = 0.47). Blood loss differed significantly across the three tertiles (*p* = 0.032), but this reflected a non-monotonic pattern (lowest in the early tertile, highest in the middle tertile, intermediate in the late tertile, Jonckheere–Terpstra test *p* = 0.87). Hospital length of stay, Textbook Outcome, conversion, and postoperative complications did not differ significantly across tertiles, although postoperative complications showed a numerically decreasing trend (20% to 10.5% to 0%) that approached significance on trend testing (logistic regression on ordered period, *p* = 0.067). Neither the composite TO nor the composite failure endpoint differed significantly across chronological tertiles (composite textbook outcome: 45.0%, 36.8%, and 47.4% with a *p* = 0.84; composite failure: 15.0%, 21.1%, and 5.3% with a *p* = 0.41), with both showing a non-monotonic pattern rather than an ordered chronological trend. Under the Delphi definition, textbook outcome was numerically higher in successive tertiles (60.0%, 68.4% and 78.9%), but this difference was not statistically significant (*p* = 0.47; trend *p* = 0.21) and no conclusion is drawn from it.

**Figure 1:**
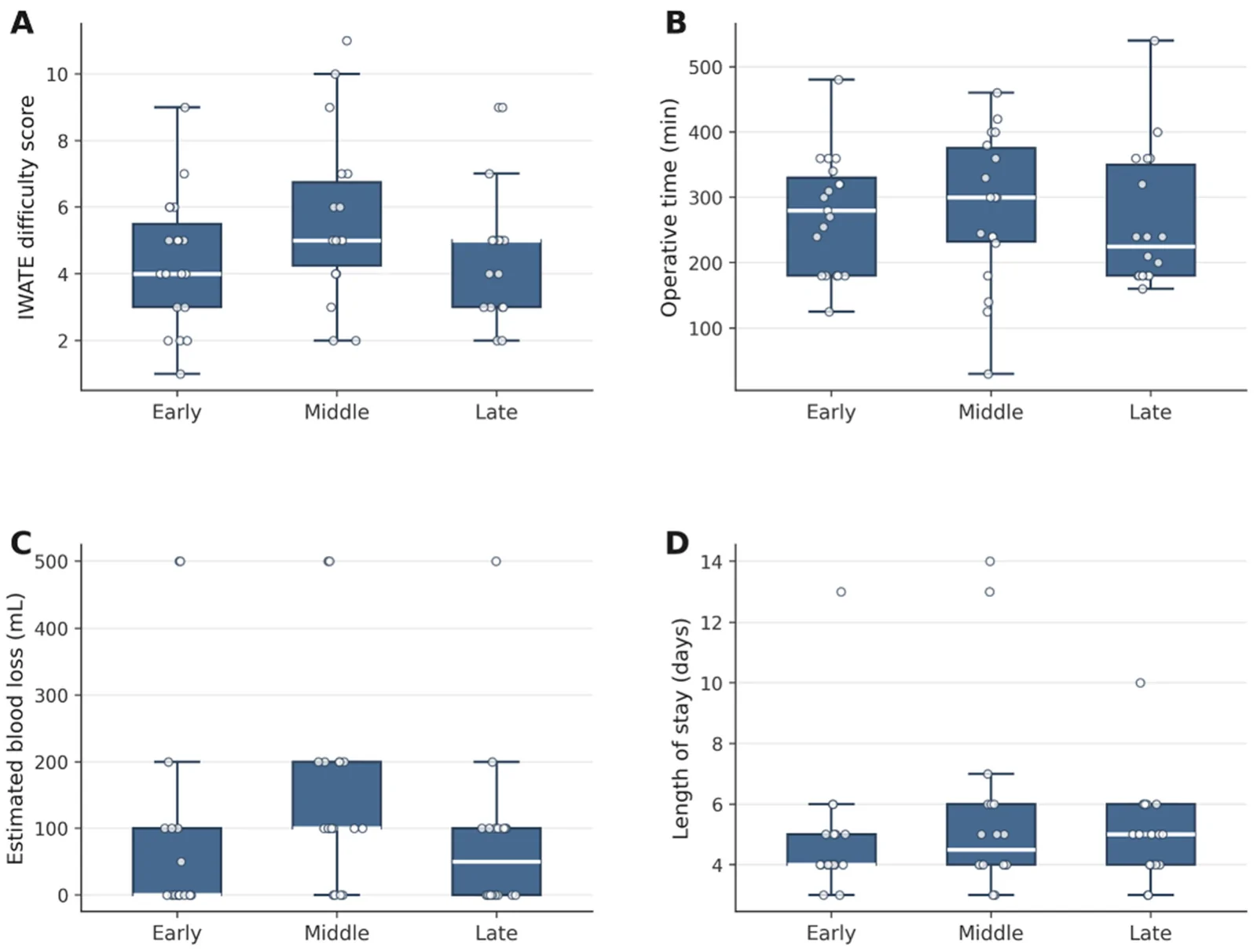
Chronological (tertile) cohort comparison of intraoperative and short-term postoperative outcomes across the case sequence. The cohort (*n* = 58) was divided into chronological tertiles according to case sequence: (**A**) IWATE difficulty score; (**B**) operative time; (**C**) estimated blood loss; (**D**) length of hospital stay. In each box plot the central line denotes the median, the box spans the interquartile range and the whiskers extend to the most extreme values within 1.5 × IQR; individual patient values are overlaid as points.

Full results are presented in Table 5 and Figure 1.

### 3.5. Learning Curve Analysis

**Figure 2:**
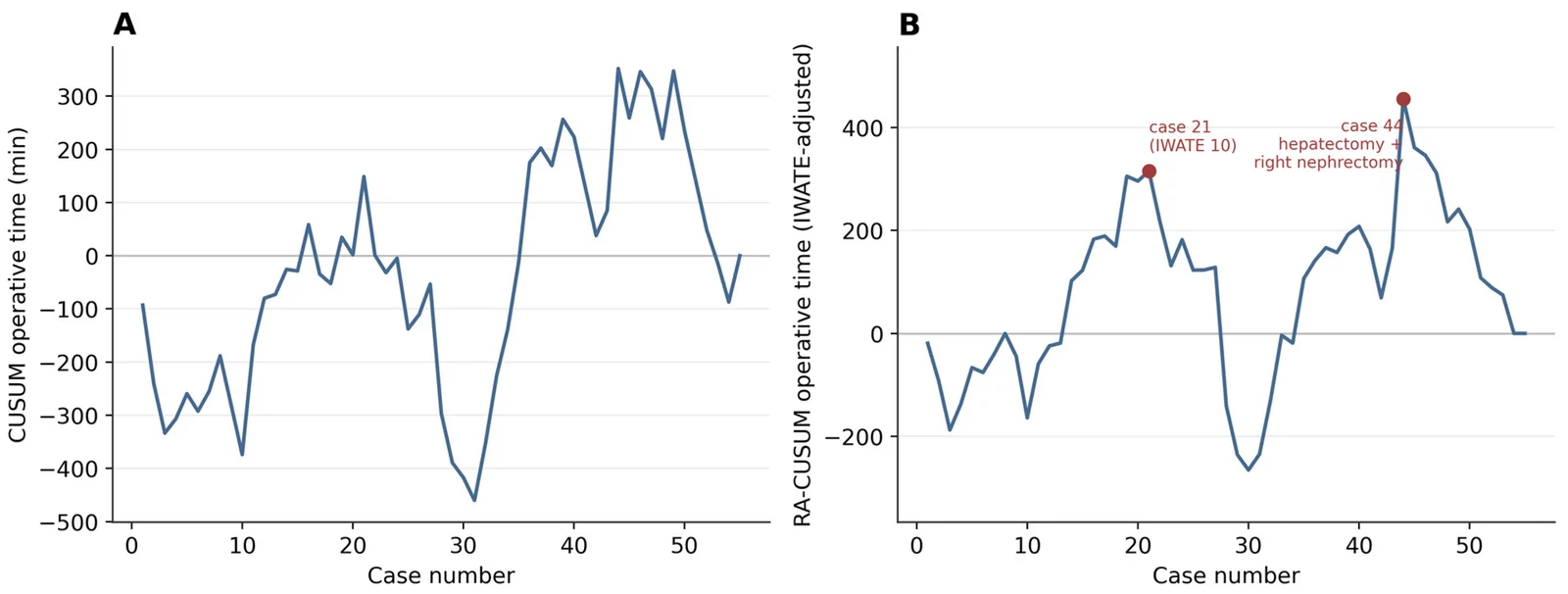
CUSUM analysis of operative time across the case sequence. (**A**) Unadjusted CUSUM and (**B**) IWATE-adjusted risk-adjusted CUSUM (RA-CUSUM) of operative time. Both curves display a non-linear, multiphase pattern without a single sustained inflection point. Case-specific extremes (highlighted) corresponded to individual high-complexity procedures—a prolonged intermediate-difficulty resection and an expert-level procedure.

Operative time was significantly associated with IWATE score on linear regression (β = 26.3 min per IWATE point, 95% CI: 15.9–36.6; R^2^ = 0.32; *p* < 0.001), whereas no association was found with chronological case order. CUSUM analysis of operative time used the cohort mean (269.7 min) as the reference value. Both the unadjusted CUSUM and the IWATE-adjusted RA-CUSUM displayed a non-linear, multiphase pattern without a sustained inflection point. Case-level review indicated that the extremes of both curves corresponded to identifiable individual procedures, most notably a left hepatic lobectomy combined with a right nephrectomy (case 44, 540 min) and an Expert-difficulty resection (case 21, IWATE 10; Figure 2).

**Figure 3:**
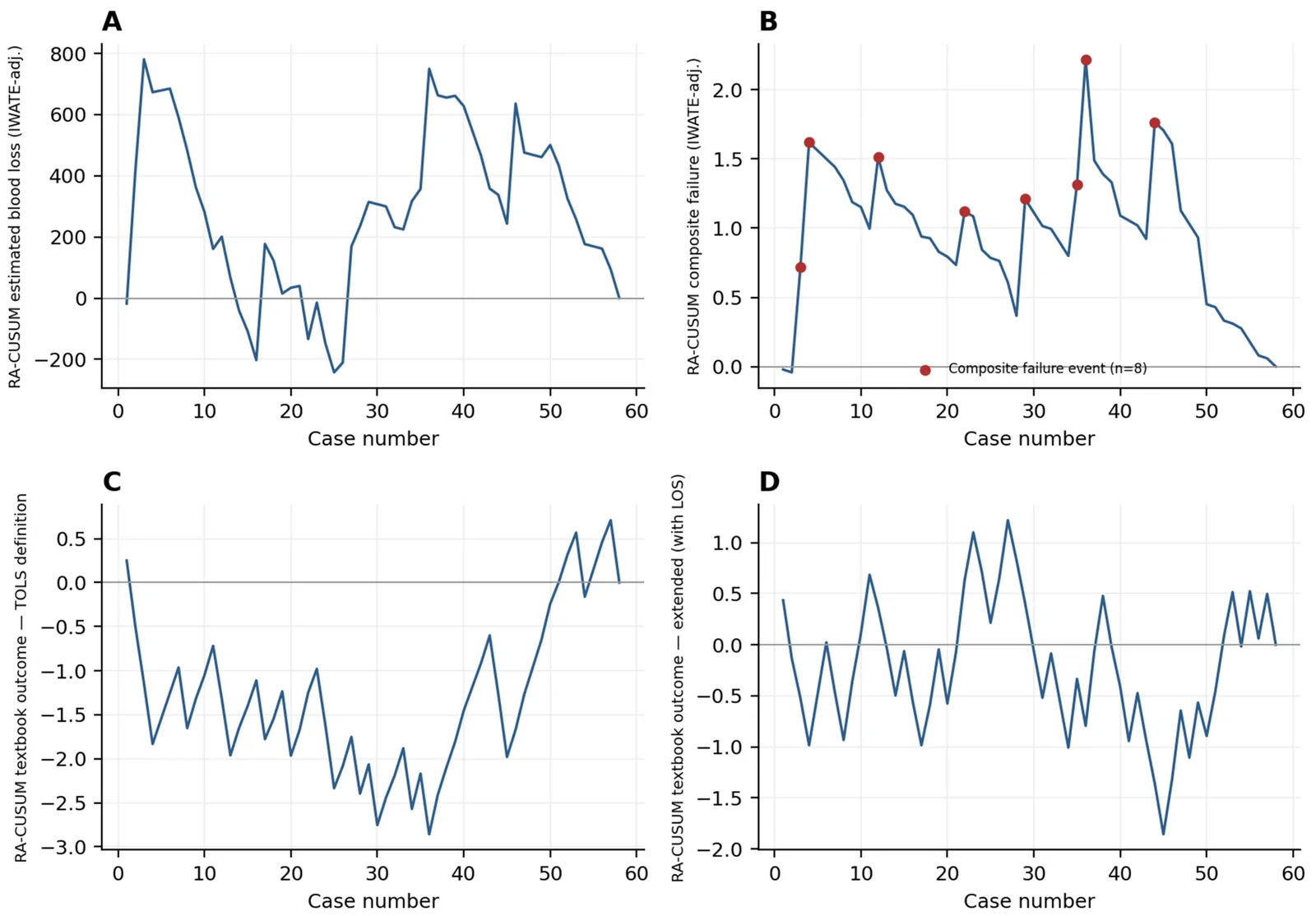
IWATE-adjusted CUSUM (RA-CUSUM) analyses across the 58 consecutive robotic liver resections. (**A**) Estimated blood loss; (**B**) composite failure endpoint (conversion, severe postoperative complications or 90-day mortality), with the eight individual events highlighted; (**C**) textbook outcome according to the Delphi (TOLS) definition; (**D**) textbook outcome according to the extended definition including length of stay.

CUSUM analysis of estimated blood loss used the cohort mean (106.0 mL) as the reference value, with the corresponding RA-CUSUM adjusted for the IWATE score. Both curves showed transient increases coinciding with a small number of outlier cases, the highest cumulative value being reached at case 3 (a mini-ALPPS with 500 mL of blood loss), without evidence of progressive worsening over time; blood loss remained low throughout the series (Figure 3A). Because only two severe complications occurred, no complication-based CUSUM could be meaningfully interpreted. For the composite failure endpoint, the IWATE-adjusted RA-CUSUM rose over the first 36 cases, reflecting seven of the eight events, with two closely spaced events at cases 35–36 producing the highest cumulative value; thereafter, despite a further event at case 44, the curve declined (Figure 3B). With eight events in total, this pattern is descriptive and cannot be interpreted as evidence of a change in performance.

Neither textbook outcome curve identified a discrete inflection point. Under the Delphi definition, the RA-CUSUM declined to a nadir at case 36 and rose thereafter to its maximum at case 57 (Figure 3C); under the extended definition, the curve oscillated around zero without a sustained direction (Figure 3D). The corresponding tertile analyses showed no significant differences (*p* = 0.47 and *p* = 1.00, respectively).

## 4. Discussion

This retrospective, single-surgeon series of the first 58 consecutive robotic liver resections demonstrates that, at an intermediate-volume center, a newly established RLR program is feasible, with perioperative safety outcomes—including TO achievement, conversion rate, and 90-day mortality—remaining stable across the study period. Interestingly, outcomes were driven primarily by procedural complexity (IWATE score) rather than chronological surgical experience. Neither the CUSUM/RA-CUSUM analyses nor chronological stratification identified a discrete, experience-dependent inflection point in operative time.

This finding departs from the classic learning-curve paradigm reported in several high-volume single-surgeon series [22], in which distinct phases of competency, proficiency, and mastery were identified after variable thresholds, and warrants specific discussion. Indeed, most published robotic liver learning-curve series describe surgeons who first acquired general robotic proficiency in another subspecialty—most commonly colorectal surgery, the most frequent entry point to robotic platforms in general surgical training—before transitioning to hepatobiliary procedures [10]. In such cases, the observed CUSUM curve is likely captures the superposition of two distinct learning processes: the time required for instrument handling and team coordination, and a procedure-specific component related to the technical demands of hepatic parenchymal transection, vascular control, and management of the cirrhotic or otherwise diseased liver. In the present series, robotic training was initiated directly in hepatobiliary surgery, without a preceding general or colorectal robotic caseload. We assume that this training pathway may have altered the shape of the observed learning curve. Furthermore, our learning curve spans over a relatively short period, in contrast with published series [22,23].

This observation, while hypothesis-generating given the retrospective, single-surgeon design, raises the possibility that direct subspecialty training in robotic hepatobiliary surgery—as opposed to sequential training via a generalist robotic caseload—may accelerate the attainment of stable, complexity-appropriate performance, particularly for surgeons who have already achieved mastery in open liver surgery, provided that case selection is carefully matched to the surgeon’s evolving robotic experience using a validated difficulty score, such as the IWATE.

The strong association between the IWATE score, operative time (*p* < 0.001) and length of stay (*p* = 0.030), in the absence of a comparable association with chronological case order, supports the construct validity of this difficulty score in a robotic hepatobiliary setting and is consistent with recent validation data from both intermediate-volume [22,23] and large multicenter robotic cohorts [7], as well as from series specifically validating the IWATE score in robotic liver resection [24,25]. Moreover, a significant decrease in TO achievement rate (*under our extended definition*) with increasing difficulty (*p* = 0.016), reinforces the rationale for IWATE-based case selection during the early phases of a robotic hepatobiliary program, rather than solely sequential, volume-based progression.

Another clinically relevant finding of this series is the consistently low rate of major morbidity and the absence of mortality across the entire study period. We acknowledge, however, that 58 procedures cannot establish the safety of the initial adoption phase, and these observations are descriptive.

No post-hepatectomy liver failure occurred, bile leak was observed in a single patient (1.7%), and only two Clavien–Dindo grade ≥ IIIa complications were recorded across 58 consecutive resections, including four major hepatectomies. Overall conversion rates accord to roughly 10% of our series and, interestingly, all events occurred in the early and middle tertile of the case sequence. Hemodynamic instability due to patient position, oncological risk, large tumor size and technical difficulty were the main reasons for conversion. These outcomes compared favorably with those reported from both intermediate-volume and considerably higher-volume robotic hepatobiliary programs (Table 6).

With regard to learning curve analysis focus falls on TO curves: the CUSUM and RACUSUM curves showed an initial phase in which global perioperative results were better than expected compared to the overall cohort average, and then a decline wherein ideal outcomes were less frequently achieved. This finding likely reflects the multifactorial nature of TO, which incorporates not only safety endpoints but also oncological, recovery-related, and process-related measures. Therefore, achieving TOs may require a longer maturation period than avoiding major adverse events alone. Additionally, when conversion is considered to be a failure in composite TO, the curve appeared more stringent and showed a deeper decline, suggesting that conversion could be a discriminator of technical proficiency in robotic liver surgery [26]. Overall, the CUSUM and RA-CUSUM curves are consistent with different domains of performance maturing at different times, although the small sample size limits these findings to descriptive and hypothesis-generating findings.

Overall, these findings suggest that the learning curve for RLR should not be interpreted as a single threshold phenomenon. Rather, different domains of performance appear to mature at different time points.

Our choice of difficulty score also warrants comment. The IWATE score was developed for laparoscopic liver resection, whereas the Tampa Difficulty Score is the first system designed specifically for robotic hepatectomy [27], and has been validated internally [28] and externally in a multicenter analysis of the AMILES registry [29]. It incorporates robot-specific determinants of difficulty that laparoscopy-derived systems do not capture. We used the IWATE score because our prospective database was constructed around its variables and because it allows for direct comparison with the majority of published minimally invasive and robotic series, including those used for benchmarking here. Nonetheless, a robotic-specific instrument may characterize procedural difficulty more appropriately in this setting, and the use of a laparoscopy-derived score is a limitation of our analysis. Prospective robotic series should ideally report both until their comparative performance in predicting outcomes is established.

### 4.1. Future Directions

Prospective continuation and follow-up of this cohort will allow for future assessment of long-term outcomes in relation to the perioperative findings reported here.

Prospective multicenter studies should re-evaluate the current standards for hepatobiliary robotic training, especially in resource-limited settings, where specific challenges inherent to the purchase and maintenance of robotic systems exist [30]. In such settings, the choice of training pathway (e.g., direct subspecialty immersion versus generalist-first progression) should be reconsidered in light of the encouraging outcomes reported here from a center where the conventional, sequential training trajectory was not pursued. Future multicentric research—encompassing both trainee- and consultant-level surgeons across centers with varying levels of robotic platform availability—is needed to define training strategies that optimize the sustainability, diffusion, and feasibility of robotic hepatobiliary surgery beyond high-volume, high-resource centers. To this end, it will be essential to continue collecting local data on the efficacy and safety of robotic surgery in these contexts to justify investments and adapt protocols to available resources.

### 4.2. Limits

This study has several limitations. First, its retrospective, single-center, single-surgeon design limits generalizability, although it is also what enables valid classical CUSUM analysis. Second, the sample size, while adequate to detect the strong association between IWATE and operative outcomes, resulted in limited statistical power within specific subgroups, notably the IWATE Expert category (*n* = 2) and major hepatectomies (*n* = 4); estimates within these subgroups should be interpreted with corresponding caution. Third, docking time was not systematically reported and extracted from operative time, which could represent a possible confounding in the learning curve analysis, as well as the performance of concurrent, non-hepatic procedures. Additionally, the case mix was heavily weighted towards minor resections (54 of 58) with only four major hepatectomies and two Expert-difficulty procedures so the findings apply to the introduction of a robotic program that predominantly performs minor resections and cannot be extended to robotic liver surgery as a whole.

## 5. Conclusions

A newly established RLR program at an intermediate-volume center is associated with favorable short-term outcomes; no mortality and a low rate of major morbidity were observed, without clustering in the early phase. Findings from learning curve trajectories argue against a discrete, case-number-defined learning threshold. Case selection remains crucial, and outcomes should be predicted based on procedural complexity, as captured by the IWATE score, rather than case order.

The study was conducted in accordance with the Declaration of Helsinki. The prospective collection and study of data from patients undergoing minimally invasive liver surgery at our institution, within the framework of the I Go MILS registry, was approved by the IRCCS ISMETT Institutional Review Board (IRRB/26/14, approved 19 May 2022). The present work is a retrospective analysis of prospectively collected data from that approved database.

## Figures and Tables

**Table 1:** Baseline demographic and clinical characteristics (*n* = 58).

Characteristic	Entire Cohort (*n* = 58)
Age, years, median (IQR)	65 (54–72)
Male patients, *n* (%)	35 (60.3)
BMI, kg/m^2^, mean ± SD	26.5 ± 5.2
ASA class, *n* (%)	
II	17 (29.3)
III	33 (56.9)
IV	8 (13.8)
Any comorbidity, *n* (%)	42 (72.4)
Charlson Comorbidity Index, mean ± SD	4.5 ± 2.8
Liver cirrhosis, *n* (%)	17 (29.3)
Child–Pugh class A among cirrhotic patients (evaluable *n* = 15)	15 (100%)
Previous abdominal surgery, *n* (%)	21 (36.2)
Portal hypertension, *n* (%)	1 (1.7)
Neoadjuvant therapy, *n* (%)	8 (13.8)

Note: Child–Pugh class could be determined in 15 of the 17 patients with cirrhosis; complete laboratory data were unavailable in the remaining two.

**Table 2:** Tumor, IWATE difficulty, and operative characteristics (*n* = 58).

Characteristic	Entire Cohort (*n* = 58)
Diagnosis, *n* (%)	
Hepatocellular carcinoma	27 (46.6)
Benign/non-oncologic	15 (25.9)
Colorectal liver metastasis	6 (10.3)
Cholangiocarcinoma	5 (8.6)
Gallbladder cancer	3 (5.2)
Breast cancer liver metastasis	1 (1.7)
PEComa	1 (1.7)
Tumor size, cm, median (IQR)	4.0 (2.5–7.0)
Number of nodules, *n* (%)	
Solitary	51 (87.9)
Bilobar distribution, *n* (%)	5 (8.6)
Vascular infiltration, *n* (%)	9 (15.5)
IWATE score, median (IQR)	5 (3–6)
IWATE difficulty category, *n* (%)	
Low (1–3)	15 (25.9)
Intermediate (4–6)	32 (55.2)
Advanced (7–9)	9 (15.5)
Expert (10–12)	2 (3.4)
Extent of resection, *n* (%)	
Minor	54 (93.1)
Major	4 (6.9)
Anatomical resection, *n* (%)	37 (63.8)
Conversion to open, *n* (%)	6 (10.3)

**Table 3:** Perioperative and postoperative outcomes of the entire cohort (*n* = 58).

Outcome	Entire Cohort (*n* = 58)
Operative time, min, median (IQR)	250 (180–360)
Estimated blood loss, mL, median (IQR)	100 (0–100)
Intraoperative transfusion, *n* (%)	0 (0)
Pringle maneuver used, *n* (%)	28 (48.3)
Pringle time, min, median (IQR), among those clamped (*n* = 28)	15 (11–47.5)
Intraoperative complication (any), *n* (%)	4 (6.9)
Hospital length of stay, days, median (IQR)	4.5 (4–6)
Any postoperative complication, *n* (%)	6 (10.3)
Clavien–Dindo grade, *n* (%)	
*0*	51 (87.9)
*I*	3 (5.2)
*II*	2 (3.4)
*IIIa*	1 (1.7)
*IIIb*	1 (1.7)
Bile leak, *n* (%)	1 (1.7)
Post hepatectomy liver failure, *n* (%)	0 (0)
Reintervention for postoperative complication, *n* (%)	1 (1.7)
90-day readmission, *n* (%)	6 (10.3)
90-day mortality, *n* (%)	0 (0)
Textbook Outcomes, TOLS definition, *n* (%)	40 (69.0)
Textbook outcomes, extended definition, *n* (%)	27 (46.6)
Composite endpoint (TO + no conversion), TOLS/extended, *n* (%)	37 (63.8)/25 (43.1)

**Table 4:** Perioperative outcomes stratified by IWATE difficulty category.

Outcome	Low (*n* = 15)	Intermediate (*n* = 32)	Advanced (*n* = 9)	Expert (*n* = 2)	*p*-Value
Operative time, min, median (IQR)	180 (160–240)	270 (180–310)	360 (345–380)	440 (420–460)	**<0.001**
Blood loss, mL, median (IQR)	0 (0–100)	100 (0–100)	200 (0–200)	50 (0–100)	0.21
LOS, days, median (IQR)	4 (3–4)	5 (4–5)	6 (5–6)	5 (4–6)	0.030
TOs, TOLS definition, *n* (%)	12 (80.0)	22 (68.8)	4 (44.4)	2 (100)	0.29
TOs, extended definition, *n* (%)	11 (73.3)	12 (38)	2 (22.2)	2 (100)	0.016
Composite TOs, TOLS, *n* (%)	12 (80.0)	21 (65.6)	3 (33.3)	1 (50.0)	0.11 ‡
Composite TOs, extended, *n* (%)	11 (73.3)	12 (37.5)	1 (11.1)	1 (50.0)	0.012 ‡
Clavien–Dindo ≥ I, *n* (%)	1 (6.7)	4 (12.5)	2 (22.2)	0 (0)	0.66 ‡
Any post-operative complication, *n* (%)	1 (6.6)	2 (6.2)	3 (33.3)	0 (0)	0.160 ‡
Conversion, *n* (%)	0 (0)	3 (9.4)	2 (22.2)	1 (50.0)	0.055 ‡
Composite failure, *n* (%)	0 (0)	4 (12.5)	3 (33.3)	1 (50.0)	**0.039 ‡**

‡ Fisher’s exact test.

**Table 5:** Perioperative outcomes according to chronological tertile of the case sequence. JT: Jonckheere–Terpstra trend test.

Outcome	Early (*n* = 20)	Middle (*n* = 19)	Late (*n* = 19)	*p*-Value	JT *p*-Trend
IWATE score, median (IQR)	4.5 (3–6)	5 (4–7)	5 (3–5)	0.40	0.89
Operative time, min, median (IQR)	275 (180–330)	300 (230–380)	210 (180–360)	0.55	0.47
Blood loss, mL, median (IQR)	0 (0–100)	100 (100–200)	0 (0–100)	0.032	0.87
LOS, days, median (IQR)	4 (4–5)	5 (4–6)	5 (4–6)	0.73	—
TOs, TOLS definition, *n* (%)	12 (60.0)	13 (68.4)	15 (78.9)	0.47	
TOs, extended definition	9 (45.0)	9 (47.4)	9 (47.4)	0.99	—
Composite TOs, TOLS	12 (60.0)	10 (52.6)	15 (78.9)	0.25	
Composite TOs, extended	9 (45.0)	7 (36.8)	9 (47.4)	0.84	—
Conversion, *n* (%)	2 (10.0)	4 (21.1)	0 (0)	0.10	—
Composite Failure (Conversion, Clavien-Dindo ≥ IIIa, 90 days Mortality)	3 (15.0)	4 (21.1)	1 (5.3)	0.41	—
Postoperative complication, *n* (%)	4 (20.0)	2 (10.5)	0 (0)	0.12	—

**Table 6:** Benchmarking of perioperative outcomes against published robotic liver resection series.

Series	*n*	Operative Time	Blood Loss (mL)	Conversion	Major Complications	90-d Mortality	LOS (Days)
Present series	58	250 (median)	100 (median)	10.3%	3.4% (2/58)	0%	4 (median)
Sijberden et al., 2024 [22]	RLR arm	190 (139–272) (median)	100 (50–280) (median)	2.7%	6.5%	1.5%	4 (3–6) (median)
Caringi et al., 2025 [7]	343	239.6 (mean)	229.5 (mean)	4.95%	4.08%	0.9%	5.8 (mean)
Pasquale et al., 2025 [18]	150	250 ± 119 (mean)	159 ± 228 (mean)	12%	10%	1.3%	7 (mean); 5 (median)

## Data Availability

The data that support the findings of this study are available on request from the corresponding author.

## Abbreviations

The following abbreviations are used in this manuscript:

**CUSUM**	Cumulative Sum
**RA-CUSUM**	Risk-adjusted Cumulative Sum
**TOs**	Textbook Outcomes
**BMI**	Body Mass Index
**ASA**	American Society of Anesthesiologists
**LOS**	Length of Stay
**ICU**	Intensive Care Unit
